# Patient satisfaction and total hip arthroplasty: a review

**DOI:** 10.1186/s42836-019-0007-3

**Published:** 2019-09-02

**Authors:** Lauren Okafor, Antonia F. Chen

**Affiliations:** 1 Frank H. Netter M.D. School of Medicine at Quinnipiac University, North Haven, CT USA; 2 Department of Orthopaedics, Brigham and Women’s Hospital, Harvard Medical School, 75 Francis Street, Boston, MA 02115 USA

**Keywords:** Patient satisfaction, Satisfaction, Total hip arthroplasty, Orthopedic surgery, Outcomes

## Abstract

Primary total hip arthroplasty (THA) has been recognized as a reliable intervention for patients with end-stage osteoarthritis. Despite several notable advances in this procedure, studies have identified at least 7% of patients who remain dissatisfied. There is no general consensus on how to measure patient satisfaction in orthopedic surgery. However, validated tools have been used in multiple studies to further investigate this problem. A comprehensive review was conducted to examine the factors associated with patient satisfaction following THA. Associations in literature included patient expectation, age, sex, pain management, patient comorbidities (medical or psychiatric that existed prior to surgery), and length of stay. The continuous collection of patient satisfaction data using validated and reliable measurement tools is necessary to improve this important patient-reported outcome after THA.

## Introduction

In the era of increased healthcare services marketing, patient satisfaction has been identified as an essential indicator for measuring the quality of care [1], quantifying value in healthcare, and gauging the overall success of medical practice. The focus on satisfaction has been shown to increase patient retention, maximize staff morale, reduce risk of malpractice suits [2], and optimize professional satisfaction. The term “patient satisfaction” was previously defined as the patient’s reaction to several aspects of their service experience [2]. This new emphasis on outcomes that matter to patients led to the development of a wide range of measurement instruments to supplement objective measures, with subjective patient views [3]. The use of patient satisfaction surveys has allowed patients to provide a more holistic evaluation of services and enlighten clinicians on various methods to refine their practice.

Patient satisfaction data can also be applied to the development of new guidelines for the identification of deficiencies, achievements, and improvements in quality of care and health service delivery [4]. In the outpatient setting, satisfaction metrics have been used by health-care organizations to determine provider compensation via pay-for-performance reimbursement models [5]. Although this has been useful for assisting physicians with gaining a better understanding of how to improve patient outcomes, patient satisfaction is a multifactorial construct [6] that can be influenced by factors unrelated to the actual quality of care. It is now understood that an optimal patient care experience is associated with higher levels of adherence to recommended prevention and treatment processes, better clinical outcomes, better patient safety within hospitals, and less health care utilization. As patient satisfaction data have become a critical component of orthopedic surgery registry data [7], clinicians must continue to closely evaluate its involvement in the patient care experience.

Patient satisfaction has been measured in multiple orthopaedic procedures, including total hip arthroplasty. THA is a common surgical procedure that improves the lives of patients with end-stage arthritis by decreasing pain, and improving motor function and mobility as measured by validated health-related outcome tools [8–12]. However, there are still patients who remain dissatisfied following this procedure due to multiple individual factors [4, 7]. Patient satisfaction is a cornerstone in healthcare that is now being used as a metric in hospital reimbursement. Due to the projected 172% increase in demand for THA procedures over the next decade [13, 14], it is critical that clinicians continue to find ways to improve patients’ experiences as it can be useful in advancing their practice, retaining and maintaining positive relationships with patients, and securing future referrals.

**Fig. 1:**
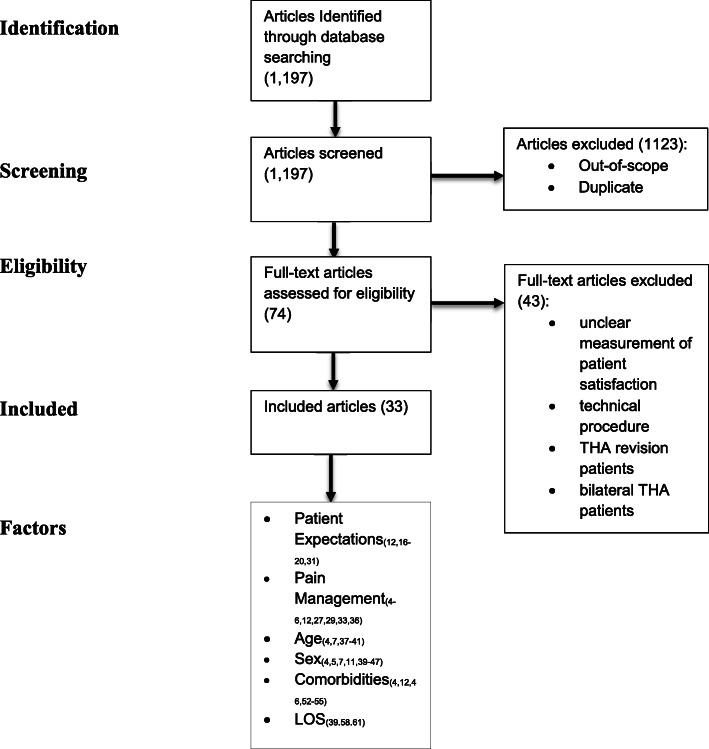
Summary of Literature Search

The purpose of this review paper is to aggregate the available literature regarding the major factors associated with THA patient satisfaction. A literature review was conducted to determine which factors have been shown to predict or influence patient satisfaction after THA. PubMed and GoogleScholar searches were performed using the terms: “patient satisfaction” with “total hip arthroplasty”, “total hip replacement (THR)”, “THA” or “THR.” Publications that were written in the English language and published between 1987 and 2018 were included. The initial search yielded 1,197 results. Once duplicate and out-of-scope articles were removed, 74 articles remained. Articles were further excluded if they did not have a clear method of measuring patient satisfaction, and if patients were undergoing bilateral staged or simultaneous THA, or revision THA. Articles discussing technical factors that could influence patient satisfaction (e.g. anterior vs. posterior approach, cemented vs. uncemented fixation, leg length discrepancy) were excluded due to potential performance or selection bias. There were a total of 33 articles that matched the above criteria. Among these articles, the associations with satisfaction included patient expectation, age, sex, pain management, patient comorbidities (medical or psychiatric that existed prior to surgery), and length of stay (LOS) that are covered in greater depth in this article (Fig. 1).

### Patient satisfaction measurement in orthopedic surgery

Currently, there is still no general consensus on the optimal method of measuring patient satisfaction [2]. The variability in interpretation of the true meaning of patient satisfaction makes the standardization of measurement by the use of reliable, valid, and meaningful metrics essential to its continued use and relevance. Although patient reported outcomes have been widely used in orthopedic surgery for several years, there has been a push to conduct more even studies that include patient satisfaction as an outcome. Presently, patient satisfaction is typically assessed in a self-reported survey format using questionnaire, numerical or Likert scales.

The Press Ganey (PG) survey, a validated patient experience evaluation that asks questions about patient’s interactions with staff, wait times, and overall assessment of their care, has one question on patient satisfaction that is only relevant to the outpatient visit [3–5]. The visual analog scale (VAS) for patient satisfaction, which has been cited as a simple, valid, and reliable method of assessing patient satisfaction, has been used after both THA and total knee arthroplasty (TKA) [6, 7]. The patient satisfaction questionnaire (PSQ) is an 8 to 18 item survey focusing on the patient’s level of satisfaction concerning the operation, their current functionality, and fulfilled expectations [4]. With respect to healthcare service marketing and referral, patients are also asked if they would undergo surgery again and if they would recommend the same operation to a friend [4]. The Hospital for Special Surgery Hip or Knee Replacement Expectations Survey contains a validated 18-item survey that has been used to measure patient satisfaction [15]. Pre-operatively, patients are asked about their expectations concerning these outcomes; postoperative follow-up questions ask patients about their overall satisfaction with the outcomes. This survey contains questions concerning patient functionality and ability to engage in daily activities (e.g., no need for assistive walking device, eliminated need for medications, improved sexual activity, and ability to exercise) [15].

With regards to the hospital experience the Hospital Consumer Assessment of Healthcare Providers and Systems Survey (HCAHPS) is a standardized instrument that has been used to capture the patient’s experience in hospital settings. This tool includes major categories asking questions about staff communication, hospital environment, pain management, and care transitions, where Never, Sometimes, Usually, and Always are the multiple-choice options. HCAHPS also asks patients for an overall hospital rating, on a scale of 0 to 10, with the increasing number indicating a better rating of satisfaction with their hospital stay. Components of the survey have been used in other studies to assess patient satisfaction after THA. For example, Mahomed et al. developed a short patient satisfaction scale for primary total joint arthroplasty using four of the HCAHPS questions to determine patient satisfaction with regards to the following: (1) pain relief, (2) ability to perform home chores, (3) improved ability to perform yard work, and (4) ability to engage in recreational activities [11]. The fifth question asked patients about their overall satisfaction with joint replacement, but this scale does not include specific items assessing satisfaction with the process of care [11]. For studies that focus on patient satisfaction after THA, most include some variations of the question, “Overall, how satisfied are you with the result of your hip replacement surgery?” [9, 10] The single question alone on overall satisfaction has been well validated as a correct indicator of patient satisfaction [15]. For most of the questionnaires mentioned, a Likert scale is used to measure patient satisfaction: [4, 11, 15] high overall satisfaction, is used as a proxy for the satisfaction outcome, and is indicated by patients who select “satisfied” or “very satisfied” to the majority of the questions on the survey. Low satisfaction is indicated by patients who mostly choose “very dissatisfied”, “dissatisfied”, or “neutral”.

There is a subtle difference between satisfaction related to the outcome of care and the process of care [9]. While these concepts may not be mutually exclusive, a patient who experiences a negative outcome of care might still report satisfaction with the process of their care. Regardless, both aspects must be assessed to form a holistic picture of patient satisfaction. As a result, patient satisfaction remains a multidimensional construct that is poorly defined in orthopaedic surgery [10]. To utilize a definition that includes the context of health care delivery and also acknowledges treatment outcomes, patient satisfaction utilized in this article will refer to contentment with ability to perform daily activities post-THA and overall satisfaction with THA (e.g. hospital stay and process of care).

### Patient expectations

Patient expectations are widely discussed in current THA outcomes research [12, 16–30], along with compliance with evidence-based guidelines. Similar to patient satisfaction, patient expectation is a complex and dynamic quality that is difficult to define, measure, and analyze [30]. Patient expectations are characterized as the anticipants that given events are likely to occur during or as a result of medical care [31]. When considering THA, expectations rely on patients’ assessment of their own disability and pain, and may also be affected by whether the surgeon recommends surgical treatment [12]. Additionally, patients can present with a wide range of expectations that are not necessarily related to pain. Two important expectations of patients undergoing THA included pain relief and improvement in physical function [12, 30]. Various methods have been used to evaluate patient expectations in orthopaedic surgery, including direct questioning, short questionnaires, and validated surveys [17].

In the past, patient expectations significantly affected patient satisfaction ratings with THA outcomes [17, 32]. The traditional, widely accepted component of this concept affirms that decreasing the discrepancy between patient expectations and the outcome of surgery was a key determinant of patient satisfaction [12]. Considering that THA is an elective procedure for patients seeking improved functionality and quality of life, the expectation would be that higher satisfaction should be reported in the absence of major post-operative complications. Mahomed et al., for example, examined patient expectations (dichotomised as high or low with respect to the likelihood of complications) [31] in predicting outcomes after total joint arthroplasty [19]. The expectation of low complication risk from THA was identified as an independent predictor of greater post-operative satisfaction at six months post-surgery, as measured on a patient satisfaction scale [19]. Using the definition of patient expectation in Uhlmann *et. al*, the expected event is low complications after THA. Therefore, if a patient leaves the hospital without any major complaints, their expectations are fulfilled, increasing the likelihood of satisfaction with the process of their care. Some current models associate higher expectations with higher satisfaction [16, 17, 20].

A prospective study by Neuprez et al. supported this with the finding that preoperative expectations, which were measured 20 days prior to surgery, were the single best positive predictor of postoperative satisfaction one year after THA [16]. Overall, the extent to which patient expectations influence patient satisfaction appears to have some potentially positive effects, with the exception of one study which associated higher expectations with lower satisfaction [18]. Therefore, it is important for surgeons to establish realistic goals and expectations with their patients with regards to postoperative outcomes after THA. This step could be instrumental in helping patients achieve appropriate levels of expectations, while providing opportunities for favorable patient-physician interaction and reducing patient’s overall perception of being unsatisfied. Ultimately, research using validated expectations tools should be implemented to further investigate this relationship.

### Pain management

Pain is the principal indication for performing THA [19, 25], and many patients can experience relief in the immediate postoperative period. Despite several advances in surgical techniques geared towards alleviating this issue, some patients continue to report dissatisfaction after total joint arthroplasty [26]. Numerous studies have explored pain management as predictors of patient satisfaction [4, 5]. In evaluation of the VAS for patient satisfaction, a high correlation between a patient’s pain score and Oxford hip score suggested that pain was one of the most important factors in patient satisfaction [5, 6]. Additional studies have also supported this claim, citing pain relief as a critical component of maximizing patient satisfaction after THA [4, 27–29]. Pre-operative pain management using nonsteroidal anti-inflammatory drugs (NSAIDs) has been associated with improved recovery [33, 34], increasing both overall satisfaction and pain satisfaction as measured by a short HCAHPs survey [33]. On the other hand, chronic pre-operative use of benzodiazepines was associated with lower patient satisfaction [33].

Another interesting finding was the negative association between increased opioid use in the post-anesthesia care unit (PACU) and patient satisfaction with pain management [33]. This finding contradicts previous researches supporting the amount of pain relief as an important component of patient expectations [12, 30], indicating that decreasing pain by the administration of opioids alone may adversely affect patients. Increased opioid intake could have a number of side effects [35] and sequelae that cause a patient to report their experiences as less than satisfactory. Mistry et al. also found a positive correlation between a patient’s post-THA perception of pain control and his/her perception of the orthopedist, nurse, and overall satisfaction [36]. These findings suggested that adequate pre- and post-operative pain management, specifically multimodal analgesia and less opioid medications, may improve patient satisfaction after THA [33].

### Age

The literature has established an association between age and clinical outcomes [10], with some variability on how age affects patient satisfaction. Some studies have found similar patient satisfaction in all age groups [4, 37–41], while other studies have reported less satisfaction in younger patients undergoing THA [7]. A possible explanation is that younger patients who may be accustomed to a more active lifestyle are more likely to be negatively affected by hip diseases [7] and have higher expectations compared to older patients undergoing total joint replacement [40]. There is evidence in the literature that older patients may experience greater satisfaction, which may be related to lower expectations of experiencing some pain relief after living with debilitating degenerative joint disease for many years. However, nationwide data collected by the Swedish Hip Arthroplasty Registry classified older age as a negative predictor for all outcomes [7], including patient satisfaction, in over 34,000 total hip replacement procedures. It is possible that there may be some confounding factors that can account for the variation seen between studies, and future research should be performed to determine specific variables associated with age and patient satisfaction after THA.

### Sex

There are many studies that discuss patient sex and THA satisfaction [4, 7, 39–45]. Outcome scores in women are often lower than in men measured by both pain and satisfaction measures in some studies [40]. One study, which used the short PSQ [11], strongly associated female sex and dissatisfaction (scaled PSQ score of < 50) with the results of primary THA [46]. Male sex has also been associated with slightly greater satisfaction, despite less improvement in pain [7]. In many studies, however, sex is often a component of secondary analysis that has no association or unclear significance in overall patient satisfaction with THA [39–44]. Anakwe et al. argued that sex could not be considered as an isolated indicator of patient satisfaction, although it is a preoperative variable [4]. Elibol et al. found no differences between male and female primary THA satisfaction at a minimum of 6 months post-operatively [44]. In both populations, patients were less satisfied with stairclimbing abilities, which was a component of their daily activities. Similarly, a study evaluating patient satisfaction at five years post-THA, found that patient satisfaction with regard to patients’ ability to do housework, participate in recreational activities, and reported improvement in quality of life, was the same between males and females [47].

While there have been some gender differences noted in the postoperative period, there have also been differences between sexes with regards to perioperative factors that strongly influenced patient satisfaction with overall hospital stay. Using post-operative Press Ganey scores, Delanois et al. found that pain management influenced overall hospital rating for men, while staff responsiveness influenced hospital ratings for women [5]. These data suggest that a gender-based focus on post-THA satisfaction may be useful for orthopedic surgeons who are interested in improving the patient experiences and subsequent satisfaction ratings.

### Comorbidities

Comorbidities are defined as patient conditions or diseases associated with the development or causation of the immediate disease of interest. Comorbidities can be diagnosed at different points in time, which may lead to different associations with adverse outcomes [48]. Previously, the Charnley classification, which primarily assesses coexisting musculoskeletal problems, was shown to predict patient satisfaction long-term functional improvement at 1 year post-THA [4, 10]. Patients were assigned into one of 3 categories: **Category A**, for patients with unilateral hip disease; **Category B**, for patients with bilateral hip disease, and **Category C**, for patients with multiple joint diseases or other major medical conditions impairing walking capacity [7]. However, this measure is less frequently used, and orthopaedic surgeons often use the Charlson Comorbidity Index (CCI) or Elixhauser Comorbidity Index (ECI) for assessing comorbidities [49, 50]. The CCI encompasses 17 comorbidities, with two subcategories that address diabetes and liver disease [49]. Each condition is assigned an integer weight from one to six, with a weight of six representing the most severe morbidity; summation of the weighted comorbidity scores results in a summary score [49]. The ECI is a more current model that covers 31 conditions, including many prevalent comorbidities that the CCI and Charnley measures do not, such as hypertension, obesity, and psychiatric disorders [50, 51].

Greene et al. found that the Charnley classification score was the strongest predictor of patient satisfaction with surgical outcomes as measured by the patient satisfaction VAS [52]. Additionally, the ECI was the only comorbidity scale that influenced patient satisfaction VAS at 1 year [52]. There were no apparent relationships between CCI and patient satisfaction in THA patients. It is reasonable to hypothesize that, in many cases, if a comorbidity contributes to severe post-THA complications, the patient may feel less satisfied with their outcome due to obstacles in recovery. On the other hand, one postoperative complication out of 850 patients who underwent THA did not predict the 7% patient dissatisfaction rate at 1 year post-THA [4]. Also, when performing THA in patients with specific comorbidities, such as hemophilia A [53–55] and diabetes [12], these patients may have higher satisfaction compared to patients without these conditions.

There is still evidence that the relationship between comorbidities and patient satisfaction depends on the number of comorbidities a patient has. The finding that patients with no comorbidities are more satisfied than patients with one or more comorbid conditions suggests an additive effect of comorbidities on declining patient satisfaction [46]. The type of comorbidity should be considered as well.

For example, a psychiatric disorder could have different relationship with patient satisfaction compared to a systemic or metabolic disorder. In general, patients with depression reported less pain reduction and satisfaction with surgical treatment [4]. Depression and somatoform disorder may explain the relatively inferior outcome in some patients after THA [56], because of its multiplicative interaction with musculoskeletal pain. Antidepressant use also interacts with this variable and is associated with less satisfaction after THA [52]. Good mental wellness is cited as a preoperative predictor of satisfaction [12], suggesting that patients with low mental wellbeing should be identified and provided with more information with regards to expectations and potential interventions [47]. Psychological factors are not routinely evaluated pre-operatively, but could arguably be useful when completing a patient’s comorbidity profile to evaluate patient reported satisfaction with their experiences and outcomes following THA.

### Length of stay

Although some studies find no association between LOS and THA [57], in general, a shorter LOS seems to play a role in patient satisfaction following many orthopedic procedures. With the implementation of fast-track surgery, LOS after THA has been reduced from 8 days to 1–2 days or even outpatient surgery. In patients undergoing unilateral THA or TKA in a fast track setting, Specht et al. reported that fast track THA patients had shorter LOS and higher overall satisfaction than their normal THA counterparts [39]. Similarly, Husted et al. also found a correlation between shorter LOS and certain aspects of satisfaction, such as pain treatment and overall stay [58], which correspond to the process of care component of patient satisfaction. A study evaluating a time-based, patient-centered fast track program for THA found that a standard LOS of 24 h did not compromise the quality of treatment or patient satisfaction [59]. Pain, dizziness, and general weakness are common clinical reasons for being hospitalized at 24 and 48 h postoperatively, and can contribute to patient discomfort and satisfaction [60]. Additionally, in patients with longer LOS and lower satisfaction ratings, the patient’s lack of knowledge concerning individual factors that could affect their LOS, such as comorbidities, age [61], and psychological factors, can lead to unrealistic expectations of how long their stay could be [36]. Improving patient education about individual factors affecting longer hospital LOS after THA may increase patient satisfaction in some populations undergoing THA [38].

## Conclusions

Multiple factors are associated with patient satisfaction following THA, which include patient expectations, pain management, age, sex, comorbidities, and length of hospital stay. For surgeons interested in improving satisfaction ratings after THA, decreasing the discrepancy between surgeon’s and patient’s expectations could provide an opportunity for the patient to better understand their likely outcomes and make more realistic goals for themselves. This requires consideration of the diverse populations of patients undergoing this procedure, and the wide range of factors related to their outcomes. Future studies evaluating these factors with validated tools will be helpful to better understand patient satisfaction after THA.

## Data Availability

The data and material for this review paper are all available in published articles.

## Abbreviations

CCI: Charlson Comorbidity Index
ECI: Elixhauser Comorbidity Index
HCAHPS: Hospital Consumer Assessment of Healthcare Providers and Systems Survey
LOS: Length of stay
NSAIDs: nonsteroidal anti-inflammatory drugs
PACU: Post-anesthesia care unit
PG: Press Ganey
PSQ: Patient satisfaction questionnaire
THA: Total hip arthroplasty
THR: Total hip replacement
TKA: Total knee arthroplasty
VAS: Visual analog scale
